# A Survey to Assess Loneliness and Predictive Factors Among Students at a US Medical School

**DOI:** 10.1007/s40596-026-02366-0

**Published:** 2026-05-28

**Authors:** Aishwarya Gautam, Jacob McCann, Ruthy Amkraut, Mireya Diaz, Erin Lamb

**Affiliations:** Case Western Reserve University School of Medicine, Cleveland, OH USA

**Keywords:** Loneliness, Medical education, Medical students

## Abstract

**Objective:**

This study examined levels of loneliness among medical students at a US medical school and identified demographic and lifestyle factors associated with increased loneliness.

**Methods:**

An anonymous cross-sectional survey using the 20-item UCLA Loneliness Scale was distributed to all medical students. The survey also collected data on demographics, living arrangements, financial limitations, extracurricular involvement, and social habits. One-way analysis of variance was used to compare mean loneliness scores across categorical variables. Multivariable linear regression assessed associations between various factors and loneliness scores, with a supplementary logistic regression serving as a robustness check.

**Results:**

A total of 194 student responses were included, reflecting a response rate of 22% after 79 surveys were excluded for quality. The mean loneliness score was 44.1 (SD = 11.1), with 43 (22%) students scoring in the low range (20–34), 89 (46%) in the moderate range (35–49), 52 (27%) in the moderately high range (50–64), and 10 (5%) in the high range (65–80). Higher loneliness scores were significantly associated with less frequent family contact, limited participation in activities due to financial constraints, and reduced socialization outside of school. Identifying as a cisgender woman was a protective factor against loneliness.

**Conclusion:**

Medical students in this sample experienced moderate levels of loneliness on average. Key predictors of higher loneliness included financial barriers to socialization, decreased family interaction, and limited social activity. Given potential academic and emotional consequences of loneliness, medical schools should consider implementing interventions that promote social support and reduce barriers to socialization.

Loneliness, which has been deemed an epidemic in the USA, is defined as “a discrepancy between one’s desired and achieved levels of social relations” and is a subjective rather than objective state [1–3]. Despite this subjectivity, loneliness has neurobiological and hormonal correlates, including altered brain network activity and increased hypothalamic-pituitary-adrenal axis activation [4, 5]. Loneliness is also associated with additional morbidity such as coronary artery disease and stroke [6].

Loneliness is both common and associated with significant health effects. It affects approximately one-third of people in industrialized nations and has been linked to worse physical and mental health in young adults [3, 7–9]. Medical training is often associated with loneliness and can coincide with a known peak in loneliness in early adulthood (i.e., late-20s) [10]. Compared to attending physicians, both medical students and physician trainees have higher rates of loneliness, with Keiner et al. reporting that 21% of medical students experience intense loneliness compared to just 9% of faculty physicians [11]. In this study, loneliness among medical trainees and attendings was associated with age ≥ 40, female gender, and non-white race [11]. Another study performed at Chicago-based medical schools found the average student reported a moderate degree of loneliness and that loneliness increased the risk of students mislabeling others’ emotional expressions [12]. While younger age was found to have a significant effect on increased loneliness, neither gender nor time in medical school was found to be significant [12]. Other research has found decreased levels of loneliness are predictive of improved academic performance [13]. While the effects of loneliness in medical students have been studied, to our knowledge, no prior study has extensively examined the risk factors for loneliness exclusively in medical students.

We conducted a survey to measure loneliness and identify risk factors among medical students at one US allopathic medical school. This survey utilizes the UCLA Loneliness 20-Item Scale to measure loneliness in medical students and examines personal life, financial situation, and participation in extracurricular activities to identify potential risk factors [14].

## Methods

The survey was administered from February to March 2024 at an allopathic medical school in the USA. The questionnaire was distributed via email to 880 medical students enrolled across all academic years, including those pursuing concurrent degrees. After the initial invitation, there were two subsequent reminders, each sent 10 days apart. The study was approved by the medical school institutional review board, and participants were informed about the study purpose and their ability to withdraw at any time without consequences. An incentive was offered in the form of a raffle for eight $25 Amazon gift cards. Inclusion criteria were current enrollment at the School of Medicine at the time of participation and return of the survey with a completed Loneliness Scale by the deadline.

The survey was administered through Qualtrics (Qualtrics, Provo, UT). All surveys were anonymous, and Qualtrics’ duplicate and response quality monitoring features were utilized to exclude duplicate surveys and straightlined responses (i.e., when a respondent picks the same response to all questions). Surveys with ≥ 80% straightlining or a duplicate score > 75 were excluded.

The survey had four sections. Section 1 included the UCLA Loneliness Scale (Version 3), a validated 20-question scale that measures responses on a 4-point Likert scale and has high reliability (Cronbach’s *α* = 0.89–0.94) [14]. The remaining sections used multiple choice, open-ended, dichotomous, and Likert-scale based questions. Section 2 collected demographics (seven items) and Sect. 3 collected personal and social factors (11 items). Section 4 collected information on engagement with peers and school organizations (15 items). Participants were further prompted to indicate how meaningful participation in each organization was to them using a Likert scale (1 = “not very”; 2 = “somewhat”; 3 = “very”; or 4 = “extremely” meaningful).

Statistical analyses were performed using Qualtrics, Excel, and XLSTAT. The primary outcome was the loneliness score as measured by the UCLA Loneliness Scale (range 20–80), treated as a continuous variable.

One-way analysis of variance (ANOVA) was used to identify variables individually associated with loneliness score. Variables assessed included age, gender identity, race, year in medical school, marital status, living situation, frequency of seeing family, financial stress, financial barriers to social participation, frequency of socialization outside of curricular activities, physical activity, and extracurricular involvement.

Variables that were significant in ANOVA were entered into a multivariable linear regression model to identify independent predictors of loneliness score. Non-significant variables were removed iteratively to reduce collinearity. For regression analyses, ordinal variables (e.g., frequency of family contact, socialization, and financial barriers) were coded numerically to reflect increasing frequency or severity. Gender identity was coded as a binary variable (cis woman = 1, cis man = 0).

As a sensitivity analysis, a multivariable logistic regression was also performed using a dichotomized loneliness outcome defined as above versus below the sample mean (> 44 vs. ≤ 44), with the same set of predictors. The model-building strategy mirrored that used for the linear regression, with all candidate predictors initially included and subsequently reduced through iterative removal of non-significant variables to obtain a final model. This analysis was conducted to assess whether the direction and statistical significance of associations observed in the linear regression were consistent when loneliness was alternatively modeled as a dichotomous outcome. Notably, responses from non-cis individuals (*n* = 4) were excluded from both regressions due to small subgroup size.

## Results

Of 880 students, 292 responded (33%). Of the 292 responses, 74 were excluded for not completing every item of the UCLA-Loneliness Scale, preventing a score from being calculated for those respondents. An additional 11 were excluded for straightlining, and 13 more were excluded for being duplicate responses. The final sample was 194 (22% of students). Four non-cisgender respondents were included in descriptive analyses but excluded from regression models due to a small group size.

Respondents were primarily young adult medical students (median age 25) across training years from varying backgrounds; most lived with peers, maintained regular family contact, reported at least some financial stress, and socialized outside required curricular activities fewer than three times per week (Table 1). On average, respondents participated in 6.5 (SD 4.83) organizations affiliated with the medical school.

**Table 1:** Demographics and variables of cohort of medical student loneliness survey respondents

	Total respondents (*N*) = 194
Age	*N* = 190
Mean (SD)	25.7 (2.4)
Median	25
Gender, % (*N*)	*N* = 192
Cis man	33 (64)
Cis woman	65 (124)
Non-binary/gender non-conforming	1 (2)
Trans man	0 (0)
Trans woman	0 (0)
Prefer not to answer	1 (2)
Race, % (*N*)	*N* = 192
White/Caucasian	58 (111)
Black	6 (11)
Asian	36 (70)
Native Hawaiian or other Pacific Islander	1 (1)
American Indian/Native American	0 (0)
Other	3 (5)
Prefer not to answer	4 (7)
Spanish, Hispanic, or Latino ethnicity, % (*N*)	*N* = 191
Yes	6 (12)
No	179
Sexual identity, % (*N*)	*N* = 192
Straight	77 (148)
Gay	4 (7)
Bisexual	10 (19)
Pansexual	1 (2)
Asexual	2 (3)
Queer	1 (2)
Prefer not to answer	6 (11)
Year of medical school, % (*N*)	*N* = 193
First year	20 (39)
Second year	31 (60)
Third year	16 (30)
Fourth year	23 (44)
PhD portion of MD/PhD	3 (6)
Research/gap year	7 (14)
Marital status, % (*N*)	*N* = 192
Never been married	79 (151)
Living with a partner	12 (23)
Married	8 (16)
Divorced/separated	1 (2)
Frequency seeing family, % (*N*)	*N* = 193
< 1 time per year	1.6 (3)
3–5 times per year	30 (57)
6 + times per year	31 (59)
Lives with them	5 (9)
Socializing outside of curriculum, % (*N*)	*N* = 192
0 days/week	8 (15)
1–2 days/week	59 (114)
3–4 days/week	26 (50)
5 +	7 (13)
Perceives financial barriers to socialization, % (*N*)	*N* = 192
Never	16 (31)
Rarely	33 (64)
Sometimes	38 (72)
Frequently	10 (20)
All of the time	3 (5)

Loneliness scores were calculated using the UCLA Loneliness Scale. The mean and median scores were 44.1 and 43.0, respectively. Among respondents, 43 (22%) had a low degree of loneliness (score 20–34), 89 (46%) had a moderate degree of loneliness (score 35–49), 52 (27%) had a moderately high degree of loneliness (score 50–64), and 10 (5%) had a high degree of loneliness (65–80) [15].

ANOVA showed that living alone (*p* = 0.01), stressing about finances (*p* = 0.04), and forgoing social activities due to financial strain (*p* = 0.04) were each individually positively associated with loneliness scores. More frequent socializing outside of curricular activities (*p* < 0.001) and seeing family more often (*p* < 0.00001) were negatively associated with loneliness scores. Marital status was also associated with loneliness scores by ANOVA (*p* < 0.00001) with married respondents having the lowest loneliness score. Variables such as age, race, physical activity, year in school, and extracurricular participation, including the number of organizations respondents participated in, were not significantly associated with loneliness in our sample.

Aforementioned factors found to be individually significant via ANOVA were included in the multivariable linear regression model. After iterative removal of non-significant variables due to collinearity, the final model retained the following significant predictors: frequency of seeing family (“seeing family”), whether finances limited participation in activities (“activities finances”), socialization, and gender identity (a binary variable with “1” representing women and “0” representing men). Table 2 presents only these significant variables; non-significant variables are not shown. Less frequent family contact was associated with a 2.49-point higher loneliness score per category decrease. Similarly, forgoing social activities due to finances more frequently and attending fewer non-school-sanctioned social events were associated with 2.25 and 5.11 higher loneliness scores per category decrease, respectively. Finally, identifying as a cis woman versus a cis man was associated with a 3.25-point lower loneliness score.

**Table 2:** Linear and logistic regression results for predictors of loneliness scores among medical students

Variable	Linear coefficient (95% CI)	*p*-value	Odds ratio (95% CI)
Seeing family less frequently	2.49 (0.96, 4.02)	0.002	1.57 (1.10, 2.25)
Financial barriers to socialization	2.25 (0.80, 3.69)	0.003	1.46 (1.04, 2.06)
Decreased frequency of socialization	5.11 (3.21, 7.02)	< 0.001	2.66 (1.60, 4.42)
Female gender	–3.25 (–6.15, –0.35)	0.028	0.62 (0.32, 1.22)
Intercept	19.10 (11.83, 26.38)	< 0.001	—

The linear regression models loneliness as a continuous variable (UCLA Loneliness Scale score, range 20–80) with a positive coefficient indicating a positive relationship between loneliness and the predictor. The linear coefficient describes the magnitude of difference in loneliness score from 1 bucket to the next (e.g., seeing family 1–3 times per year versus 3–5 times per year). The logistic regression, presented as a sensitivity analysis, models the odds of reporting an above-sample-mean loneliness score (> 44) versus ≤ 44, reflected in the odds ratios. Only variables with statistically significant associations are shown

*CI*, confidence interval

The logistic regression also found that “seeing family,” “activities finances,” and “socialization” were significantly associated with above-average loneliness scores. However, gender identity was not significant in this regression. Similar to the linear regression, “socialization” was the most impactful variable, with those who socialized less often having a higher chance (odds ratio = 2.66) of having an above-average loneliness score (Table 2).

## Discussion

We quantified loneliness among students at a midwestern medical school using the UCLA Loneliness Scale [14]. The mean score for all respondents (*N* = 194) was 44.1 on a 20–80-point scale, corresponding to moderate feelings of loneliness based upon widely used UCLA Loneliness Score categorization [15]. Seeing family, financial limits on social engagement, socialization frequency, and gender identity were significant predictors. Level of socialization was the most impactful of these factors, with spending zero days socializing instead of 1 day resulting in a 5.11 higher loneliness score on average (Table 2: linear coefficient). Some variables—marital status, for instance—were individually significantly correlated with loneliness in ANOVA but no longer significant in a linear regression. This indicates that marital status was not an independent predictor of loneliness in our sample; the contribution of marital status to loneliness was likely better explained by one of the remaining significant variables.

To our knowledge, this is the first study focused on risk factors for loneliness in medical students. Smith et al. found an average loneliness score of 38.03 to 39.42, which is lower than our cohort’s average [12]. This study was administered before the COVID-19 pandemic, and studies have shown that loneliness increased during the pandemic, including among students, pointing to one possible explanation [16]. Keiner et al. found that over half of medical students reported feeling lonely at least some of the time, with 21% reporting feelings of intense loneliness [11]. We found that 32% of students had a moderately high or high degree of loneliness on the UCLA Loneliness Scale. Direct comparisons with this study are challenging, as Keiner et al. used a screening tool rather than a validated scale of individuals’ loneliness like the UCLA Loneliness Scale.

We found that identifying as a cisgender woman was a protective factor against increased loneliness, whereas Smith et al. demonstrated no significant effect of gender on loneliness score and Keiner found that female physicians reported more severe loneliness [11, 12]. Studies of the population at large have shown that loneliness is higher in men than in women [17]. These findings suggest that female physicians (versus female medical students or non-medical professionals) may experience unique factors predisposing them to increased loneliness.

Given our findings, medical schools should consider interventions that promote non-academic socialization as a tangible way to minimize loneliness among its students. Schools might offer qualifying students a daily meal card, for instance, to help them meet their basic needs, thus giving them more freedom to use remaining funds on whatever social activities they desire. Providing financial assistance to help students travel to see their families could also help offset loneliness. These interventions should complement, not replace, existing well-being services, such as counseling programs, access to a nutritionist, and initiatives that promote adequate sleep, physical activity, and help-seeking behavior.

While we did not measure depression or anxiety, prior research has demonstrated that loneliness is strongly correlated with anxiety, depression, and suicidal ideation [9, 18]. Loneliness has also been linked to decreased quality of one’s relationships [19]. Future studies should include validated measures of depression and anxiety to better characterize the relationship between loneliness and these conditions among medical students.

Limitations of this study include using data from a single institution and a 22% effective response rate, which introduces the potential for significant selection bias. For instance, it is possible that the study is more likely to include individuals for whom loneliness is a concern. Nevertheless, loneliness scores show a broad distribution, with 22% in the low range. Results are also limited by underrepresentation of first- and third-year students. Third-years likely responded less due to clinical demands, while first-year nonresponse may reflect survey unfamiliarity or enhanced support making a loneliness survey feel less relevant. Moreover, the sample was relatively homogenous, with an overrepresentation of white, female, and cisgender students, making claims about the association with race and gender identity potentially misleading. The small number of married respondents (*n* = 8) may also have influenced results, as marital status was significant in univariate analysis but not in multivariable models. Furthermore, this study did not assess other potentially important contributors to loneliness, such as clinical experiences, mentorship, spirituality, or experiences of discrimination. Finally, although logistic regression was performed as a sensitivity analysis, the dichotomous cutoff (> 44 vs. ≤ 44) was derived from the sample mean and does not correspond to established UCLA Loneliness Scale categories. As such, these findings should be interpreted as supportive of, rather than equivalent to, the primary linear regression results. Additional studies are needed to improve generalizability, particularly among more diverse student populations, including those with non-cisgender identities.

In conclusion, we found that the average medical student in our sample reported experiencing feelings of moderate loneliness, with a mean score of 44.1. Less frequent family contact, decreased socialization due to financial stress, and reduced socialization outside of school were associated with increased levels of loneliness, while identifying as a cis woman was associated with less loneliness in our cohort. Additional, larger studies are necessary to better represent medical students and to characterize their loneliness. Medical schools seeking to reduce loneliness in their students may attempt to organize financially subsidized non-academic social activities for their students.

## Data Availability

Please contact Dr. Erin Lamb at egl36@case.edu for data requests. De-identified data is available as permitted by IRB-approved protocol.
